# Association of killer cell immunoglobulin-like receptors and their cognate HLA class I ligands with susceptibility to acute myeloid leukemia in Iranian patients

**DOI:** 10.1038/s41598-023-38479-x

**Published:** 2023-07-15

**Authors:** Sara Mirzazadeh, Peyman Bemani, Hossein Halimi, Mohammad Nabi Sanaee, Narges Karami, Mani Ramzi, Shirin Farjadian

**Affiliations:** 1 Department of Immunology, Shiraz Medical School, Shiraz University of Medical Sciences, Shiraz, Iran; 2 Department of Immunology, School of Medicine, Isfahan University of Medical Sciences, Isfahan, Iran; 3 Department of Hematology and Medical Oncology, Namazi Hospital, Shiraz University of Medical Sciences, Shiraz, Iran

**Keywords:** Immunogenetics, Haematological cancer

## Abstract

Acute myeloid leukemia (AML) is one of the most prevalent leukemia in adults. Among the various NK receptors, killer immunoglobulin-like receptors (KIRs) carry out indispensable roles in NK cell development and function through engaging with class I human leukocyte antigens (HLA-I) as their ligands. Besides divergent KIR and HLA loci, KIR**/**HLA-I combinations have a significant effect on NK cell response. In this case–control study, we aimed to verify the association of KIR**/**HLA-I combinations with susceptibility to AML in the Southwestern Iranian population. KIR and HLA genotyping was performed with PCR-SSP by some novel primers for 181 patients with AML and 181 healthy controls. According to our results, the frequencies of KIR3DS1 (*p* = 0.0001, OR = 2.32, 95% CI 1.51–3.58), KIR2DS4fl (*p* = 0.02, OR = 1.53, 95% CI 1.05–2.21), CxT4 genotypes (*p* = 0.03, OR = 2.0, 95% CI 1.05–3.82), and T4 gene cluster (*p* = 0.01, OR = 1.99, 95% CI 1.17–3.41) were significantly higher in patients than controls, while C1/C2 genotype (*p* = 0.00002, OR = 0.39, 95% CI 0.25–0.61), HLA-A Bw4 (*p* = 0.02, OR = 0.6, 95% CI 0.38–0.94), and HLA-A*11 (*p* = 0.03, OR = 0.57, 95% CI 0.34–0.95) alleles were more frequent in controls. In addition, inhibitory (i)KIR**/**HLA-I combinations analysis revealed higher frequencies of KIR2DL1( +)**/**HLA-C2( +), KIR2DL2/3( +)**/**HLA-C1( +), KIR3DL1( +)**/**HLA-A Bw4( +), and KIR3DL2( +)**/**HLA-A*03/11( +) in the control group (*p* = 0.002, OR = 0.49, 95% CI 0.3–0.78; *p* = 0.04, OR = 0.62, 95% CI 0.39–0.99; *p* = 0.04, OR = 0.63, 95% CI 0.4–0.99; and *p* = 0.03, OR = 0.62, 95% CI 0.4–0.95, respectively). Overall, the number of iKIR**/**HLA-I combinations was more in the control group. Moreover, KIR3DS1( +)**/**HLA-B Bw4^Ile80^( +) and the sum of HLA-B Bw4/A Bw4 combined with KIR3DS1 as activating KIR**/**HLA-I combinations were more frequent among patients than controls (*p* = 0.01, OR = 1.99, 95% CI 1.14–3.49 and *p* = 0.005, OR = 1.97, 95% CI 1.22–3.19, respectively). In conclusion, our results postulate that inhibitory combinations play a protective role against AML by developing potent NK cells during education. It is noteworthy that KIR**/**HLA-I combination studies can be applicable in donor selection for allogeneic NK cell therapy in hematological malignancies.

## Introduction

Acute myeloid leukemia (AML) is one of the most prevalent kinds of leukemia in adults^1,2^. It is characterized by a disorder in myeloid differentiation, increased proliferation, and accumulation of immature cells in bone marrow, circulation, and other tissues^3^. Two classifications systems have been presented for AML. The French-American-British (FAB) classification system is based on morphological and cytochemical criteria. The World Health Organization (WHO) classification considers genetic abnormalities and evidence of dysplasia in addition to morphology and differentiation of cells^4,5^.

NK cells have essential roles in preventing leukemic cell establishment and progression^6–8^. Different receptors exist on the surface of these cells, which transmit inhibitory or activating signals^9^. Killer immunoglobulin-like receptors (KIRs) play a predominant role in NK cell development and function^10,11^. Based on the intracellular length of the KIR tails, these receptors are divided into two groups: inhibitory (i) KIRs and activating (a) KIRs. Receptors with long tails exert inhibitory signals through their ITIMs (immunoreceptor tyrosine-based inhibition motif), while those with short tails transduce activating signals through DAP12 adaptor protein, which harbor ITAM (immunoreceptor tyrosine-based activation motif)^11,12^.

The KIR gene cluster is situated inside the leukocyte receptor complex (19q13.4)^12^. There are two KIR haplotypes based on KIR gene content: “A” haplotypes contain five iKIRs (2DL3, 2DL1, 2DL4, 3DL1, and 3DL2) and one aKIR (2DS4)^13^. In addition to the full-length form (fl), KIR2DS4 has a truncated variant with a 22-bp deletion at exon five (del), which produces a secretory molecule with no known function^14,15^. However, "B" haplotypes mainly possess aKIRs (2DL2, 2DL5, 2DS1, 2DS2, 2DS3, 2DS5, and 3DS1). Both haplotypes have four framework genes (3DL3, 3DP1, 2DL4, and 3DL2)^13^. Individuals can be AA or Bx (AB or BB) genotypes according to their inherited haplotypes. KIR haplotypes are divided into centromeric and telomeric regions via a rearrangement site^16^. A cluster of four genes (2DS2, 2DL2, 2DL5B, 2DS3) is located in the centromeric half of B haplotypes determined as C4, while the T4 cluster (3DS1, 2DL5A, 2DS5, 2DS1) is placed in telomeric half. So, individuals who have all these genes of both regions are determined as the C4T4 genotype. If at least one of the centromeric genes is absent, along with the presence of all genes in the telomeric half, it is termed CxT4 despite C4Tx being related to the existence of only centromeric cluster genes entirely. Meanwhile, if one gene is missing from each cluster, it is called CxTx^17^.

Human leukocyte antigens class I (HLA-I) molecules are the most important ligands for the KIRs. NK cell development and response strongly depend on KIR**/**HLA-I interaction^18^. HLA-C alleles encode two forms of ligands based on amino acid residues at positions 77 and 80 in their α1 domains. HLA-C1 has serine and aspartic acid, but HLA-C2 has aspartic acid and lysine at these positions, respectively. Therefore, they can interact with different receptors via these residues. HLA-C1 interacts with KIR2DL2/3, and HLA-C2 ligates with KIR2DL1^11,19^. HLA-B alleles are also classified into Bw4- and Bw6-containg motifs, and just the former group can bind to KIR3DL1. The affinity of Bw4-bearing allotypes to KIR3DL1 is influenced by a dimorphic site at amino acid position 80 (Isoleucine or Threonine), as HLA-Bw4^Ile80^ binds to the receptor with greater affinity^20–22^. The Bw4 motif is also found in HLA-A*23/24/32 alleles, called HLA-A Bw4^23,24^. Furthermore, HLA-A*03/11 allotypes can interact with KIR3DL2^25^.

Although the extracellular domains of some aKIRs have high sequence homology with some iKIRs (2DS1-2DL1, 2DS2-2DL2, and 3DS1-3DL1 pairs), little information is available about the ligands of aKIRs, and their ligands are mostly unknown except for KIR2DS1 which binds to HLA-C2^26,27^. Recently, it has been found that some HLA-I ligands can also bind to aKIRs but with lower affinity than the corresponding iKIRs (Supplementary Fig. S1)^11^.

Concerning the importance of KIR/HLA-I interaction in regulating NK cell education^28^ and function, this study was designed to investigate the association of KIR genes and their cognate HLA-I ligands with susceptibility to AML in the Southwestern Iranian population.

## Results

To investigate the combined effect of KIR and their cognate HLA-I ligands on susceptibility to AML, KIR and HLA genotyping was applied for 181 patients, and the results were compared with 181 healthy controls. Patients with an average age of 49.94 ± 16.91 years (range 17–87) included 71 women (50.18 ± 18.1 years) and 110 men (49.79 ± 16.17 years).

### Frequencies of KIR genes and genotypes in patients with AML and controls

The frequency of KIR genes is shown in Table 1. As shown, only the frequency of KIR3DS1 was significantly higher in patients than in controls (*p* = 0.0001, OR = 2.32, 95% CI 1.51–3.58). KIR2DS4 fl allele and fl/fl genotype were more common in patients than controls (*p* = 0.02, OR = 1.53, 95% CI 1.05–2.21 and *p* = 0.03, OR = 2.04, 95% CI 1.03–4.07, respectively) (Supplementary Table S1). Our data showed no significant difference in AA and Bx genotypes between patients and controls; however, the CxT4 genotype and T4 gene cluster were more frequent in patients compared to controls (*p* = 0.03, OR = 2, 95% CI 1.05–3.82 and *p* = 0.01, OR = 1.99, 95% CI 1.17–3.41, respectively) (Table 2). In total, a higher frequency of aKIRs ≥ 4 was detected in the patient group than in the control group, although this difference was not significant.

**Table 1:** Frequency of KIR genes in patients with AML compared to controls.

KIR genes	AML: n = 181 (%)	Control: n = 181 (%)	P value OR (95% CI)
**A haplotype-associated KIRs**			
3DL1	167 (92.3)	171 (94.5)	0.39
2DL1	177 (97.8)	181 (100)	0.13^‡^
2DL3	157 (86.7)	153 (84.5)	0.54
2DS4	172 (95.0)	175 (96.7)	0.42
**B haplotype-associated KIRs**			
2DS1	81 (44.8)	69 (38.1)	0.20
2DS2	103 (56.9)	111 (61.3)	0.39
2DS3	66 (36.5)	79 (43.6)	0.16
2DS5	64 (35.4)	56 (30.9)	0.37
3DS1	90 (49.7)	54 (29.8)	**0.0001**
			**2.32 (1.51–3.58)**
2DL2	108 (59.7)	109 (60.2)	0.91
2DL5	102 (56.4)	102 (56.4)	> 0.99

The Chi-square test was done based on a 2 × 2 contingency table, *p* < 0.05 was considered significant. *OR:* odds ratio, *CI:* confidence interval

^‡^p-value with Yates’ correction.

Significant values are in bold.

**Table 2:** Distribution of KIR genotypes in patients with AML compared to controls.

KIR genotypes	AML: n = 181 (%)	Control: n = 181 (%)	P-value OR (95% CI)
AA	39 (21.6)	42 (23.2)	0.70
Bx	142 (78.4)	139 (76.8)	
**Bx genotype subsets**	**n = 142 (%)**	**n = 139 (%)**	
C4T4	18 (12.7)	12 (8.6)	0.27
C4Tx	36 (25.4)	46 (33.1)	0.15
CxT4	31 (21.8)	17 (12.2)	**0.03**
			**2 (1.05–3.82)**
CxTx	57 (40.1)	64 (46)	0.31
C4 cluster	54 (38.1)	58 (42)	0.52
T4 cluster	49 (34.5)	29 (20.8)	**0.01**
			**1.99 (1.17–3.41)**

The Chi-square test was done based on a 2 × 2 contingency table, *p* < 0.05 was considered significant. *OR:* odds ratio, *CI:* confidence interval.

Significant values are in bold.

KIR profiles of patients and healthy individuals are shown in Supplementary Table S2. We found 93 different KIR gene profiles with known patterns, of which 28 were patient-specific, 38 were control-specific, and 27 profiles were identified in both groups.

### Frequencies of HLA-I ligands in patients with AML and controls

According to our results (Table 3), there was no significant difference between HLA-C allotypes (C1 and C2), HLA-B allotypes (Bw4 and Bw6), and HLA-B Bw4 isoforms (Bw4^Ile80^ and Bw4^Thr80^) between patients and controls. While our data showed a higher frequency of HLA-A Bw4 in the control group than in patients with AML (p = 0.02, OR = 0.6, 95% CI 0.38–0.94) which seems to be due to the difference of A*23/24 as a part of HLA-A Bw4. Also, HLA-A*11 was more prevalent in controls (p = 0.03, OR = 0.57, 95% CI 0.34–0.95).

**Table 3:** Frequency of HLA-I alleles as KIR ligands in patients with AML compared to controls.

HLA alleles	AML: 2n = 362 (%)	Control: 2n = 362 (%)	P-value OR (95% CI)
C1	180 (49.7)	176 (48.6)	0.76
C2	182 (50.3)	186 (51.4)	
B Bw4	150 (41.4)	145 (40.1)	0.70
B Bw6	212 (58.6)	217 (59.9)	
	**n = 181 (%)**	**n = 181 (%)**	
B Bw4^Ile80^	92 (50.8)	91 (50.3)	0.91
B Bw4^Thr80^	29 (16.0)	29 (16.0)	> 0.99
A Bw4	50 (27.6)	70 (38.7)	**0.02**
			**0.60 (0.38–0.94)**
A*23/24	37 (20.4)	53 (29.3)	0.051
A*32	18 (9.9)	20 (11.0)	0.73
Total Bw4	134 (74.0)	136 (75.1)	0.80
A*03	33 (18.2)	40 (22.1)	0.35
A*11	32 (17.7)	49 (27.1)	**0.03**
			**0.57 (0.34–0.95)**

The Chi-square test was done based on a 2 × 2 contingency table, *p* < 0.05 was considered significant. *OR*: odds ratio, *CI:* confidence interval.

Significant values are in bold.

Patients were mostly homozygous for C1 and C2 (*p* = 0.01, OR = 1.83, 95% CI 1.15–2.93 and *p* = 0.03, OR = 1.61, 95% CI 1.02–2.55, respectively) while C1/C2 heterozygote genotype was more common in the control group (*p* = 0.00002, OR = 0.39, 95% CI 0.25–0.61). Also, our results revealed no significant difference in HLA-B, HLA-A Bw4, and HLA-A*03/11 genotypes between patients and controls (Table 4).

**Table 4:** Distribution of HLA genotypes in patients with AML compared to controls.

HLA genotypes	AML: n = 181 (%)	Control: n = 181 (%)	P-value OR (95% CI)
**HLA-C genotypes**			
C1/C1	62 (34.3)	40 (22.1)	**0.01**
			**1.83 (1.15–2.93)**
C1/C2	56 (30.9)	96 (53.0)	**0.00002**
			**0.39 (0.25–0.61)**
C2/C2	63 (34.8)	45 (24.9)	**0.03**
			**1.61 (1.02–2.55)**
**HLA-B genotypes**	**n = 181 (%)**	**n = 181 (%)**	
Bw4/Bw4	36 (19.9)	33 (18.2)	0.68
Bw4/Bw6	78 (43.1)	79 (43.6)	0.91
Bw6/Bw6	67 (37.0)	69 (38.1)	0.82
**HLA-A Bw4 genotypes**	**n = 50 (%)**	**n = 70 (%)**	
A*23/24( +)/A*32( +)	5 (10.0)	3 (4.3)	0.38^‡^
A*23/24( +)/A*32(–)	32 (64.0)	50 (71.4)	0.38
A*23/24(–)/A*32( +)	13 (26.0)	17 (24.3)	0.83
HLA-A*03/11 genotypes	**n = 59 (%)**	**n = 79 (%)**	
A*03( +)/A*11( +)	27 (45.8)	30 (38.0)	0.35
A*03( +)/A*11(–)	6 (10.2)	10 (12.7)	0.65
A*03(–)/A*11( +)	26 (44.1)	39 (49.4)	0.53

The Chi-square test with Yates’ correction was done based on a 2 × 2 contingency table, *p* < 0.05 was considered significant. *OR:* odds ratio, *CI:* confidence interval.

^‡^p-value with Yates’ correction.

Significant values are in bold.

### Frequencies of KIR/HLA-I combinations in patients with AML and controls

The frequencies of iKIRs and their cognate HLA-I ligand combinations are summarized in Table 5. According to our data, the frequency of the KIR2DL1( +)**/**HLA-C2( +) combination was significantly higher in the control group than in patients (*p* = 0.002, OR = 0.49, 95% CI 0.31–0.78), whereas the frequency of the KIR2DL1( +)**/**HLA-C2(–) combination showed a significant increase in patients compared to controls (*p* = 0.01, OR = 1.83, 95% CI 1.15–2.93). There was also a similar trend for both states of the KIR2DL2/3**/**HLA-C1 combination (in the presence of the ligand: *p* = 0.04, OR = 0.62, 95% CI  0.39–0.99 and in the absence of the ligand: *p* = 0.02, OR = 1.68, 95% CI 1.06–2.68). Moreover, although the frequency of the KIR3DL1( +)**/**HLA-A Bw4( +) combination was higher in the control group (*p* = 0.04, OR = 0.63, 95% CI 0.4–0.99), the KIR3DL1( +)**/**HLA-A Bw4(–) combination was significantly more frequent in patients (*p* = 0.03, OR = 1.56, 95% CI 1.02–2.5). The frequency of the KIR3DL2( +)**/**HLA-A*03/11( +) combination was also significantly higher in the control group than in patients (*p* = 0.03, OR = 0.62, 95% CI 0.4–0.95), while the presence of the receptor in the absence of its ligands was more common in patients (*p* = 0.03, OR = 1.6, 95% CI 1.04–2.45).

**Table 5:** Distribution of iKIRs and their cognate HLA-I ligands in patients and controls.

Inhibitory KIR/HLA-I ligand			
	AML: n = 181 (%)	Control: n = 181 (%)	P-value OR (95% CI)
KIR2DL1( +)/HLA-C2( +)	115 (63.5)	141 (77.9)	**0.002**
			**0.49 (0.3–0.78)**
KIR2DL1( +)/HLA-C2(–)	62 (34.3)	40 (22.1)	**0.01**
			**1.83 (1.15–2.93)**
KIR2DL1(–)/HLA-C2( +)	4 (2.2)	0	0.13^‡^
	**n = 181 (%)**	**n = 179 (%)**	
KIR2DL2/3( +)/HLA-C1( +)	118 (65.2)	134 (74.9)	**0.04**
			**0.62 (0.39–0.99)**
KIR2DL2/3 ( +)/HLA-C1(–)	63 (34.8)	43 (24.0)	**0.02**
			**1.68 (1.06–2.68)**
KIR2DL2/3(–)/HLA-C1( +)	0	2 (1.1)	0.47^‡^
	**n = 171 (%)**	**n = 176 (%)**	
KIR3DL1( +)/HLA-B Bw4^Ile80^( +)	88 (51.5)	86 (48.9)	0.62
KIR3DL1( +)/HLA-B Bw4^Ile80^(–)	79 (46.2)	85 (48.3)	0.69
KIR3DL1(–)/HLA-B Bw4^Ile80^( +)	4 (2.3)	5 (2.8)	0.96^‡^
	**n = 171 (%)**	**n = 173 (%)**	
KIR3DL1( +)/HLA-B Bw4^Thr80^( +)	25 (14.6)	27 (15.6)	0.79
KIR3DL1( +)/HLA-B Bw4^Thr80^(–)	142 (83.0)	144 (83.2)	0.96
KIR3DL1(–)/HLA-B Bw4^Thr80^( +)	4 (2.3)	2 (1.2)	0.67^‡^
	**n = 170 (%)**	**n = 175 (%)**	
KIR3DL1( +)/HLA-A Bw4( +)	47 (27.6)	66 (37.7)	**0.04**
			**0.63 (0.4–0.99)**
KIR3DL1( +)/HLA-A Bw4(–)	120 (70.6)	105 (60.0)	**0.03**
			**1.60 (1.02–2.5)**
KIR3DL1(–)/HLA-A Bw4( +)	3 (1.8)	4 (2.3)	0.96^‡^
	**n = 175 (%)**	**n = 179 (%)**	
KIR3DL1( +)/HLA Bw4( +)	126 (72.0)	128 (71.5)	0.91
KIR3DL1( +)/HLA Bw4(–)	41 (23.4)	43 (24.0)	0.89
KIR3DL1(–)/HLA Bw4( +)	8 (4.6)	8 (4.5)	0.96
	**n = 181 (%)**	**n = 181 (%)**	
KIR3DL2( +)/HLA-A*03/11( +)	59 (32.6)	79 (43.6)	**0.03**
			**0.62 (0.4–0.95)**
KIR3DL2( +)/HLA-A*03/11(–)	122 (67.4)	102 (56.4)	**1.60 (1.04–2.45)**

The Chi-square test was done based on a 2 × 2 contingency table, *p* < 0.05 was considered significant. *OR:* odds ratio, *CI:* confidence interval.

^‡^p-value with Yates’ correction.

Significant values are in bold.

Furthermore, as shown in Table 6, KIR3DS1( +)**/**HLA-B Bw4^Ile80^( +) was the only activating combination that had a higher frequency in patients (*p* = 0.01, OR = 1.99, 95% CI 1.14–3.49). Considering the sum of HLA-A and HLA-B alleles containing the Bw4 motif in combination with their related aKIR (3DS1), the frequency of KIR3DS1( +)**/**HLA-Bw4( +) was significantly more in patients than the control group (*p* = 0.005, OR = 1.97, 95% CI 1.22–3.19), whereas the presence of HLA-B Bw4^Ile80^, HLA-B Bw4^Thr80^, and HLA-A Bw4 in the absence of their common activating receptor (KIR3DS1) was higher in the control group (*p* = 0.0004, OR = 0.4, 95% CI 0.24–0.67; *p* = 0.01, OR = 0.38, 95% CI 0.18–0.80 and *p* = 0.001, OR = 0.38, 95% CI 0.21–0.69, respectively). Also, based on our results, a higher frequency of 3 or more iKIR/HLA-I combinations was found in the control group compared with patients (Table 7).

**Table 6:** Distribution of aKIRs and their cognate HLA-I ligands in patients and controls.

Activating KIR/HLA-I ligands			
	AML: n = 148 (%)	Control: n = 156 (%)	P-value OR (95% CI)
KIR2DS1( +)/HLA-C2( +)	52 (35.1)	54 (34.6)	0.92
KIR2DS1( +)/HLA-C2(–)	29 (19.6)	15 (9.6)	**0.01**
			**2.29 (1.17–4.47)**
KIR2DS1(–)/HLA-C2( +)	67 (45.3)	87 (55.8)	0.06
	**n = 151 (%)**	**n = 168 (%)**	
KIR2DS2( +)/HLA-C1( +)	70 (46.4)	79 (47.0)	0.90
KIR2DS2( +)/HLA-C1(–)	33 (21.9)	32 (19.0)	0.53
KIR2DS2(–)/HLA-C1( +)	48 (31.8)	57 (33.9)	0.68
	**n = 134 (%)**	**n = 119 (%)**	
KIR3DS1( +)/HLA-B Bw4^Ile80^( +)	48 (35.8)	26 (21.8)	**0.01**
			**1.99 (1.14–3.49)**
KIR3DS1( +)/HLA-B Bw4^Ile80^(–)	42 (31.3)	28 (23.5)	0.16
KIR3DS1(–)/HLA-B Bw4^Ile80^( +)	44 (32.8)	65 (54.6)	**0.0004**
			**0.40 (0.24–0.67)**
	**n = 104 (%)**	**n = 76 (%)**	
KIR3DS1( +)/HLA-B Bw4^Thr80^( +)	15 (14.4)	7 (9.2)	0.29
KIR3DS1( +)/HLA-B Bw4^Thr80^(–)	75 (72.1)	47 (61.8)	0.14
KIR3DS1(–)/HLA-B Bw4^Thr80^( +)	14 (13.5)	22 (28.9)	**0.01**
			**0.38 (0.18–0.80)**
	**n = 117 (%)**	**n = 96 (%)**	
KIR3DS1( +)/HLA-A Bw4( +)	23 (19.7)	28 (29.2)	0.10
KIR3DS1( +)/HLA-A Bw4(–)	67 (57.3)	26 (27.1)	**0.00001**
			**3.60 (2.01–6.44)**
KIR3DS1(–)/HLA-A Bw4( +)	27 (23.1)	42 (43.8)	**0.001**
			**0.38 (0.21–0.69)**
	**n = 158 (%)**	**n = 150 (%)**	
KIR3DS1( +)/HLA Bw4( +)	66 (41.8)	40 (26.7)	**0.005**
			**1.97 (1.22–3.19)**
KIR3DS1( +)/HLA Bw4(–)	24 (15.2)	14 (9.3)	0.11
KIR3DS1(–)/HLA Bw4( +)	68 (43.0)	96 (64.0)	**0.0002**
			**0.42 (0.26–0.67)**

The Chi-square test was done based on a 2 × 2 contingency table, *p* < 0.05 was considered significant. *OR:* odds ratio, *CI:* confidence interval. Significant values are in bold.

**Table 7:** Number of KIR genes and KIR/HLA-I combinations in patients with AML compared to controls.

No. of KIR genes	AML: n = 181 (%)	Control: n = 181 (%)	P-value OR (95% CI)
≥ 4 aKIRs	85 (46.9)	67 (37.0)	0.055
≥ 4 iKIRs	123 (67.9)	119 (65.8)	0.65
iKIRs > aKIRs	102 (56.4)	116 (64.1)	0.13
aKIRs > iKIRs	39 (21.5)	28 (15.5)	0.13
**No. of KIR/HLA combination**			
≥ 3 aKIRs/HLA	17 (9.4)	21 (11.6)	0.49
≥ 3 iKIRs/HLA	102 (56.4)	140 (77.4)	**0.00002**
			**0.37 (0.23–0.59)**
iKIRs/HLA > aKIRs/HLA	150 (82.9)	161 (89.0)	0.09
aKIRs/HLA > iKIRs/HLA	3 (1.7)	2 (1.1)	0.99^‡^
iKIRs/HLA = aKIRs/HLA	28 (15.5)	18 (9.9)	0.11

The Chi-square test was done based on a 2 × 2 contingency table, *p* < 0.05 was considered significant. *OR:* odds ratio, *CI:* confidence interval.

^‡^p-value with Yates’ correction.

Significant values are in bold.

## Discussion

NK cells play crucial roles in immune surveillance and cancer defense. In fact, a balance between transduced signals through inhibitory and activating receptors shapes NK cell responses. Based on their structural landmark, KIRs exert a principal effect on eliciting NK cell response. It is worth noting that iKIRs have a key role in NK cell education through engagement with their endogenous cognate HLA-I ligands. Due to the independent segregation of KIR and HLA genes, molecular interactions of KIR-HLA vary among individuals based on their HLA alleles and KIR gene contents^29^. In recent years, studies have been done on the effect of KIRs and their cognate HLA-I ligands on susceptibility to various malignancies. However, there are limited data in the field of hematologic malignancies, especially AML.

In this study, 11 KIR genes and 9 HLA-I allotypes as their corresponding ligands were investigated by PCR-SSP method in patients with AML in comparison to healthy controls. Our results showed an increased frequency of aKIRs, aKIR**/**HLA-I combinations, and decreased frequency of HLA-I ligands in patients with AML. While in the control group, an elevated frequency of HLA-I ligands and iKIR**/**HLA-I combinations was observed.

Our data imply raised AML incidence in men in comparison with women. It is confirmed by American Cancer Society (ACS) information and Hematological Malignancy Research Network (HMRN)^30,31^. In fact, the different incidence is related to a non-identical immune system between the two genders.

The average age of patients with AML at the time of diagnosis, according to ACS and HMRN, was about 68 years. However, the median age of our patients was about 50 years, which is more comparable to Brazilian (less than 60 years) and Saudi Arabian (53 years) patients^32,33^. The age variation of patients among different populations might be due to their genetic origins and environmental factors^34,35^.

In our study, KIR3DS1 frequency was significantly higher in patients than in controls, and we suggested 3DS1 as a risk factor for AML. Misra et al. also reported a higher frequency of aKIRs, especially KIR3DS1among their patients with childhood ALL^36^. Unlike our study, Shahsavar et al. showed a lower frequency of 3DS1 in patients with AML, but it was not significant^37^. In contrast, Varbanova et al. reported a significantly lower frequency of 3DS1 in patients with AML^38^. Also, the 2DS4fl allele and fl/fl genotype were significantly increased among our patients, while Giebel et al. found a higher frequency of the 2DS4del allele in their patients with CML compared to the control group^39^. Furthermore, our results revealed that the T4 cluster (KIR3DS1-2DL5-2DS1-2DS5) and CxT4 genotype were significantly more frequent in patients than in controls. Therefore, the telomeric gene cluster with more aKIRs can be considered a risk factor for AML. These results describe an activating trend of NK response in patients with AML.

Moreover, we found that although there was no significant difference between patients and controls based on the frequency of HLA-C allotypes, C1/C1 and C2/C2 genotypes were more frequent in patients, and C1/C2 genotype was dominant in the control group. It seems C1/C2 genotype has heterozygote advantage and plays a protective role in the control group^40^. Indeed, the heterogenicity led to more variety of HLA-I ligands and KIR/HLA-I combinations in the control group. Similarly, no association was found between HLA-C alleles and leukemia in Hispanic/non-Hispanic and Bulgarian patients^31,38^. However, a study in the Thai population reported the low frequency of HLA-C2 and C1/C1 genotypes in patients with AML^41^. Whereas studies in the Iranian and German populations stated contradictory results and introduced HLA-C2 as a susceptibility factor to ALL^37,42^.

In terms of HLA-B allotypes (Bw4 and Bw6), genotypes (Bw4/Bw4, Bw6/Bw6, and Bw4/Bw6) and Bw4 isoforms (Bw4^Ile80^ and Bw4^Thr80^), there was no significant difference between two groups. Unlike our results, Shahsavar et al. indicated a higher frequency of HLA-B Bw4^Ile80^ in patients with AML. They also found no significant difference in HLA-A Bw4 between patients and controls^37^. Middleton et al. also observed a higher frequency of HLA-B Bw4^Ile80^ in patients with AML. They also considered the Bw4/Bw4 genotype as a predisposing and the Bw6/Bw6 genotype as a protective factor in Turkish white CML patients^43^. In contrast, the study on the Bulgarian population showed a higher frequency of HLA-B Bw4^Thr80^ in myeloid leukemia patients and the Bw4/Bw4 genotype in leukemia patients^38^.

To investigate KIR**/**HLA-I combinations, we considered different states, including (1) the presence of the receptor and ligand simultaneously, (2) the presence of the receptor in the absence of the ligand, and (3) the presence of the ligand in the absence of the receptor.

We first investigated the combination between iKIRs and their cognate HLA-I ligands. Our results showed an elevated frequency of the KIR2DL1( +)**/**HLA-C2( +) combination in the control group, while the KIR2DL1( +)**/**HLA-C2(–) combination was more frequent in patients. In other words, compared to the control group, who carried a double positive combination, patients mostly had the inhibitory receptor in the absence of its cognate ligand. Against our results, Shahsavar et al. indicated a higher frequency of KIR2DL1( +)**/**HLA-C2( +) among patients with ALL^37^.

We detected the same trend in the KIR2DL2/3**/**HLA-C1 combination. Double positives form of this combination was common among the control group, but KIR2DL2/3( +)**/**HLA-C1(–) revealed a higher frequency in our patient group.

Also, we identified an increased frequency of the KIR3DL1( +)**/**HLA-A Bw4( +) combination among the control group, but KIR3DL1( +)**/**HLA-A Bw4(–) was more common in the AML group. Moreover, the same trend was also found in the KIR3DL2**/**HLA-A*03/11 combinations. Consistent with our results, Varbanova et al. suggested a higher frequency of KIR3DL1( +)**/**HLA-A*03/11(–) among patients with myeloid leukemia, whereas Shahsavar et al. indicated an increased frequency of the KIR3DL1( +)**/**HLA-A Bw4( +) in their patients with AML^37,38^.

We next analyzed the presence of aKIRs and their cognate HLA-I ligands. Our results revealed a higher frequency of KIR2DS1( +)**/**HLA-C2(–) in the AML group. However, Misra et al. demonstrated an increased frequency of KIR2DS1( +)**/**HLA-C2( +) among children with ALL^36^.

We detected a higher frequency of KIR3DS1( +)**/**HLA-B Bw4^Ile80^( +) in the patient group, but Shahsavar et al. found this combination with lower frequency among their patients with AML^37^.

Our results demonstrated that higher frequency of 3DS1 in the absence of HLA-A Bw4, while Varbanova et al. reported a lower frequency of KIR3DS1( +)**/**HLA-A Bw4(–) in their patients with AML^38^. We also investigated the sum of all HLA-A and HLA-B alleles carrying the Bw4 motif in combination with KIR3DS1. Our results showed a higher frequency of KIR3DS1( +)**/**HLA-Bw4( +) in AML. However, the presence of ligands in the absence of their related receptor was more abundant in the control group.

Overall, our results revealed a boost in the frequency of iKIRs and their cognate HLA-I ligands in the control group but a higher frequency of iKIRs in the absence of their ligands in AML. We also observed a higher frequency of aKIR**/**HLA-I combinations in the AML group and the presence of HLA-I ligands in the absence of their corresponding aKIRs in the control group. To explain these results, we propose two different models. First, based on higher frequencies of aKIRs and aKIR**/**HLA-I combinations in patients, it seems that their NK cells are hyperresponsive and become exhausted through challenging with malignant cells. These NK cells are not able to eradicate malignant cells, but they cause chronic inflammation in the tumor microenvironment. This can be considered a reason for the delayed onset of AML, as most initial diagnoses are made between 50 and 70 years. Second, which is more plausible, NK cells represent an essential actor in immunosurveillance. To become more efficient, these cells go through licensing process in which just those NK cells that recognize HLA-I ligands by their inhibitory receptors gain the ability to respond against cancerous cells. In this connection, our results indicated a higher frequency of iKIR**/**HLA-I combinations in the control group; therefore, it seems that these licensed NK cells are more eligible for early removal of cancer cells in the control group.

To make inferences, we implied a higher frequency of KIR3DS1, KIR2DS4fl, CxT4 genotypes, and T4 genes cluster beside a higher frequency of aKIRs in patients with AML. Also, our data showed higher frequencies of C1/C2 genotype, A Bw4, and A*11 alleles in the control group; therefore, we suggest these elements as a protective factor. To summarize the results of KIR**/**HLA-I combinations, the presence of iKIRs with their cognate ligands, such as KIR2DL1( +)**/**HLA-C2( +), KIR2DL2/3( +)**/**HLA-C1( +) and KIR3DL2( +)**/**HLA-A*03/11( +) were more frequent in the control group. In contrast, the presence of these iKIRs without their cognate ligands had a higher frequency in patients with AML. Furthermore, aKIR**/**HLA-I combinations, such as KIR3DS1( +)**/**HLA-B Bw4^Ile80^( +) and KIR3DS1( +)**/**HLA-Bw4( +), were more prevalent in patients, while the presence of HLA-I ligands (B Bw4^Ile80^, B Bw4^Thr80^, and A Bw4) without their corresponding receptor, KIR3DS1, revealed an increased frequency in controls. In fact, regarding the lower frequency of iKIRs**/**HLA-I in patients, their NK cells were not properly educated and would not be sufficiently competent for inhibiting cancer cell development.

It should not be ignored that NK cell response is influenced by many different factors, such as various signals from different kinds of receptors as well as malignant cell status. Indeed, our study is a piece of the puzzle in NK function against malignant cells, and more study is necessary for drawing a whole picture of NK cell activity in cancer. Nevertheless, our study opens a new avenue for understanding the role of KIR**/**HLA-I combinations in hematologic malignancies and would be helpful in alloreactive NK cell therapy in the future.

## Methods

### Study populations

According to the WHO and FAB classifications, 181 patients with AML were included in our study from Namazi Hospital affiliated with Shiraz University of Medical Sciences (SUMS) after receiving written informed consent. The protocol of this study was designed based on the Declaration of Helsinki and approved by the SUMS Ethics Committee (IR.SUMS.REC.1400,528). A total of 75 blood samples were collected from newly diagnosed patients with AML from April 2021 to April 2022 and 106 samples were included from our previous study^44^. Due to their low white blood cell counts and low DNA yield, patients undergoing intensive chemotherapy were omitted from the study. Demographic information was collected from patients’ medical records. As the control group, DNA samples from 181 sex/ethnicity-matched healthy adults were collected from the same geographic region, Fars province in southwestern Iran.

### KIR and HLA genotyping

Genomic DNA was extracted from 300 µl of whole blood samples using the non-enzymatic salting-out method, and the concentration was adjusted at 30 ng/μl^45^.

KIR genes (2DL1, 2DL2, 2DL3, 2DL5, 2DS1, 2DS2, 2DS3, 2DS4, 2DS5, 3DL1 and 3DS1) were genotyped by polymerase chain reaction with sequence-specific primers (PCR-SSP) method using 11 specific primer pairs designed by Vilches et al.^46^. Moreover, an additional pair of primers based on the sequences reported by Ashuri et al. were used to discriminate fl and del variants of KIR2DS4^47^. DNA samples with known KIR patterns were gifted from the Shiraz Institute for Cancer Research as controls.

Genotyping of HLA-I genes was also done by PCR-SSP using 9 specific primer pairs, four of which, including HLA-C1, C2, Bw4^Ile80^ and Bw4^Thr80^ primer pairs, were before used by Tajik et al., while HLA-A*03, A*11, A*23/24/32 and Bw6 were designed in this study (Table 8)^48^.

**Table 8:** Primer sequences and PCR conditions for genotyping of HLA-I ligands by PCR-SSP method.

		Primer sequences (5ʹ─3ʹ)	PCR conditions	Amplicon size	Ref
1	HLA-A*03	F: CACGGAATGTGAAGGCCCAG R: CGTGGGGGATGAGGGGTCC	95 °C/3 min 10 cycles: 94 °C/20 s, 72 °C-62°C/30 s (− 1 °C per cycle) and 72 °C/30 s 20 cycles: 94 °C/20 s, 62 °C/30 s and 72 °C/30 s 72 °C/5 min	129	New
2	HLA-A*11	F: CACGGACGGGCCAGGTG R: CCCTCCAGGTAGGCTCTCT	95 °C/3 min 9 cycles: 94 °C/20 s, 72 °C-63°C/30 s (− 1 °C per cycle) and 72 °C/30 s 21 cycles: 94 °C/20 s, 63 °C/30 s and 72 °C/30 s 72 °C/5 min	410	New
3	HLA-A*23/24	F: GGTCTCAGCCACTCCTCGT R: GCTCTGGTTGTAGTAGCGGA	95 °C/3 min 12 cycles: 94 °C/20 s, 72 °C-60°C/30 s (− 1 °C per cycle) and 72 °C/30 s 18 cycles: 94 °C/20 s, 60 °C/30 s and 72 °C/30 s 72 °C/5 min	288	New/Tajik^48^
4	HLA-A*32	F: GGTGAGTGCGGGGTCGT R: GCTCTGGTTGTAGTAGCGGA		393	New/Tajik^48^
5	HLA-B Bw6	F: GAGCGAGGGGACCGCAG R: TGTAGTAGCCGCGCAGGT	95 °C/3 min 8 cycles: 94 °C/20 s, 72 °C-64°C/30 s (− 1 °C per cycle) and 72 °C/30 s 22 cycles: 94 °C/20 s, 64 °C/30 s and 72 °C/30 s 72 °C/5 min	344	Tajik^48^/Hong^49^

F: forward primer, R: reverse primer.

Regarding the importance of HLA-A*03 and A*11 interaction with KIR3DL2 and the lack of specific primers for detecting these HLA alleles with PCR-SSP, we designed two novel primer pairs. In addition, the previously designed HLA-A Bw4 specific primers identify not only HLA-A*23/24/32 alleles but also HLA-A*25, which has been proven not to interact with KIR3DL1 as a ligand^23,24^. Therefore we designed two specific forward primers (Table 8), which in combination with the previously published reverse primer, can specifically amplify HLA-A*23/24/32 alleles^48^. To discriminate Bw4 from Bw6-bearing allotypes and due to the lack of appropriate Bw6-specific pair of primers, we optimized Bw6-specific primer pair by combining Tajik's Bw4^Ile80^ forward primer with Hong's Bw6 reverse primer^48,49^. For designing the new primers, sequences of certain HLA alleles were extracted from the IPD-IMGT/HLA database (ftp://ftp.ebi.ac.uk/pub/databases/ipd/imgt/hla/). Then BioEdit version 7.2, CLC sequence viewer version 7.8.1, and AlleleID version 7.7 software were used to design and evaluate the thermodynamic properties and the possibility of forming secondary structures. Also, the Primer-BLAST was employed to check the specificity of the primers.

DNA samples with known HLA alleles – typed by high-resolution method – were included as controls from our previous study, which was performed in collaboration with the National Reference Laboratory for Histocompatibility, Geneva, Switzerland^50^.

Each PCR reaction contained a couple of specific primes and a pair of primers for amplifying a non-polymorphic fragment of the human HLA-DRα gene as the internal control. The negative control was a PCR mixture without template DNA, and DNA samples with known KIR and HLA genotypes confirmed by high-resolution methods were used as the positive control. Each PCR reaction was performed in a total volume of 10 μl containing 5 μl master mix (2x) including Taq DNA polymerase (Ampliqon A/S, Denmark), 0.5 μM of each specific primer, 0.1 μM of each internal control primer, and 30 ng of genomic DNA. The temperature profiles of previously designed primers were applied according to Tajik’s study, and the thermal conditions of newly designed primers are summarized in Table 8.

PCR products of different HLA and KIR genes were verified by agarose gel electrophoresis (2%) containing DNA-safe stain, and a 50 bp DNA ladder was loaded in every gel. To differentiate between 2DS4 full length (fl) and deleted (del) alleles, electrophoresis was done on 4% agarose gel. After KIR typing, genotypes of each sample were determined based on an allele frequency database (http://www.allelefrequencies.net/kir6001a.asp).

### Statistical analysis

The frequency of genes, genotypes, and KIR**/**HLA-I combinations between patients and healthy controls was compared using the Chi-square test based on a 2 × 2 contingency table. To prevent overestimation of statistical significance for small data, Yates’ correction was used when at least one cell of the 2 × 2 contingency table had a count smaller than 5. Data were analyzed using SPSS 26, and *p* < 0.05 was considered statistically significant. The odds ratio (OR) and 95% confidence interval (CI) were calculated to investigate the significance of the association of KIR genes and their cognate HLA-I ligands with susceptibility to AML.

## Supplementary Information

Supplementary Figure S1.

Supplementary Table S1.

Supplementary Table S2.

## Data Availability

The data that support the findings of this study are available from the corresponding author upon reasonable request.
