# Supplementary figures and images for: Association of killer cell immunoglobulin-like receptors and their cognate HLA class I ligands with susceptibility to acute myeloid leukemia in Iranian patients
Source: Sci Rep. 2023 Jul 15;13:11456. doi: 10.1038/s41598-023-38479-x (PMC10349836; doi:10.1038/s41598-023-38479-x)

**Supplementary Figure S1:** KIRs and their cognate HLA-I ligands (Created with BioRender.com)
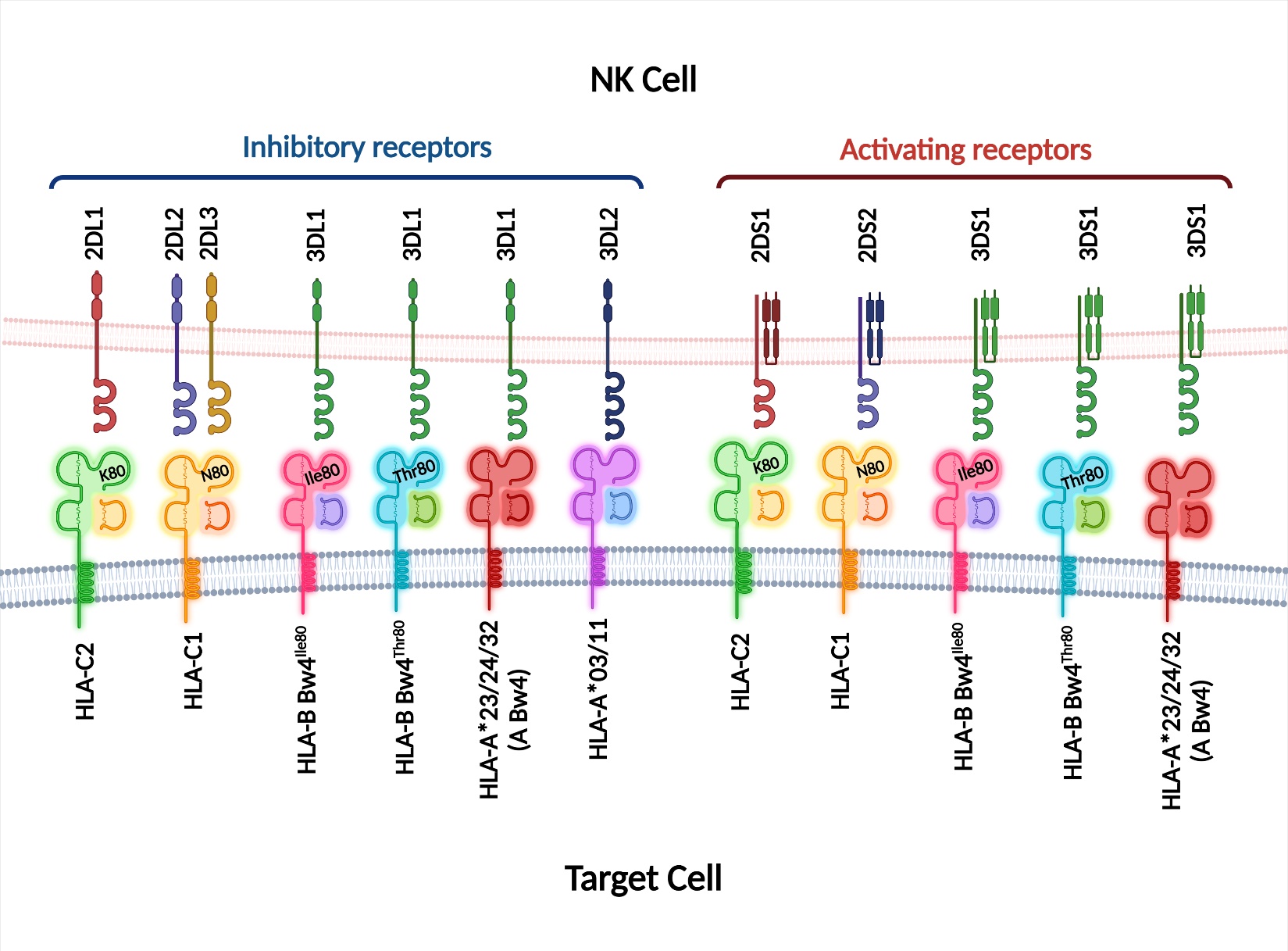
.

Supplement: Supplementary file 1 — Supplementary Figure S1. [file 41598_2023_38479_MOESM1_ESM.docx]
