# Supplementary material for: Association of killer cell immunoglobulin-like receptors and their cognate HLA class I ligands with susceptibility to acute myeloid leukemia in Iranian patients
Source: Sci Rep. 2023 Jul 15;13:11456. doi: 10.1038/s41598-023-38479-x (PMC10349836; doi:10.1038/s41598-023-38479-x)
Supplement: Supplementary file 2 — Supplementary Table S1. [file 41598_2023_38479_MOESM2_ESM.docx]

| KIR2DS4 | AML  n=172 (%) | Control  n=175 (%) | P-value  OR (95% CI) |
| --- | --- | --- | --- |
| Genotypes | | | |
| *del/del* | 114 (66.3) | 128 (73.1) | 0.16 |
| *del/full* | 32 (18.6) | 33 (18.9) | 0.95 |
| *full/full* | 26 (15.1) | 14 (8.0) | **0.03**  **2.04 (1.03-4.07)** |
| Alleles | **n=344 (%)** | **n=350 (%)** |  |
| *full* | 84 (24.4) | 61 (17.4) | **0.02**  **1.53 (1.05-2.21)** |
| *del* | 260 (75.6) | 289 (82.6) | **0.65 (0.45-0.94)** |

**Supplementary Table S1:** Allele and genotype frequencies of KIR2DS4 in patients with AML compared to controls. The Chi-square test was done based on a 2×2 contingency table, *p*<0.05 was considered significant. CN: control, OR: odds ratio, CI: confidence interval.
