# Supplementary material for: Association of killer cell immunoglobulin-like receptors and their cognate HLA class I ligands with susceptibility to acute myeloid leukemia in Iranian patients
Source: Sci Rep. 2023 Jul 15;13:11456. doi: 10.1038/s41598-023-38479-x (PMC10349836; doi:10.1038/s41598-023-38479-x)
Supplement: Supplementary file 3 — Supplementary Table S2. [file 41598_2023_38479_MOESM3_ESM.docx]

| **Genotype_ID** | **Haplogroup** | **Bx subset** | **A haplotype associated** | | | | **B haplotype associated** | | | | | | | **Framework** | | | **Pseudogenes** | | **Frequency** | |
| --- | --- | --- | --- | --- | --- | --- | --- | --- | --- | --- | --- | --- | --- | --- | --- | --- | --- | --- | --- | --- |
|  |  |  |  |  |  |  |  |  |  |  |  |  |  |  |  |  |  |  | **AML patients** | **Controls** |
|  |  |  | **3DL1** | **2DL1** | **2DL3** | **2DS4** | **2DL2** | **2DL5** | **3DS1** | **2DS1** | **2DS2** | **2DS3** | **2DS5** | **2DL4** | **3DL2** | **3DL3** | **2DP1** | **3DP1** |  |  |
| 1 | *AA* | *--* |  |  |  |  |  |  |  |  |  |  |  |  |  |  |  |  | 39 | 40 |
| 2 | *Bx* | *CxT4* |  |  |  |  |  |  |  |  |  |  |  |  |  |  |  |  | 13 | 4 |
| 3 | *Bx* | *CxT4* |  |  |  |  |  |  |  |  |  |  |  |  |  |  |  |  | 7 | 7 |
| 4 | *Bx* | *CxTx* |  |  |  |  |  |  |  |  |  |  |  |  |  |  |  |  | 17 | 16 |
| 5 | *Bx* | *C4Tx* |  |  |  |  |  |  |  |  |  |  |  |  |  |  |  |  | 10 | 16 |
| 6 | *Bx* | *C4T4* |  |  |  |  |  |  |  |  |  |  |  |  |  |  |  |  | 12 | 6 |
| 7 | *Bx* | *C4Tx* |  |  |  |  |  |  |  |  |  |  |  |  |  |  |  |  | 5 | 4 |
| 8 | *Bx* | *CxTx* |  |  |  |  |  |  |  |  |  |  |  |  |  |  |  |  | 3 | 0 |
| 9 | *Bx* | *CxTx* |  |  |  |  |  |  |  |  |  |  |  |  |  |  |  |  | 1 | 3 |
| 10 | *Bx* | *CxTx* |  |  |  |  |  |  |  |  |  |  |  |  |  |  |  |  | 1 | 0 |
| 11 | *Bx* | *C4Tx* |  |  |  |  |  |  |  |  |  |  |  |  |  |  |  |  | 1 | 5 |
| 12 | *Bx* | *CxT4* |  |  |  |  |  |  |  |  |  |  |  |  |  |  |  |  | 0 | 2 |
| 13 | *Bx* | *C4Tx* |  |  |  |  |  |  |  |  |  |  |  |  |  |  |  |  | 3 | 1 |
| 14 | *Bx* | *CxTx* |  |  |  |  |  |  |  |  |  |  |  |  |  |  |  |  | 0 | 2 |
| 18 | *Bx* | *CxT4* |  |  |  |  |  |  |  |  |  |  |  |  |  |  |  |  | 2 | 0 |
| 19 | *Bx* | *CxTx* |  |  |  |  |  |  |  |  |  |  |  |  |  |  |  |  | 1 | 1 |
| 20 | *Bx* | *CxTx* |  |  |  |  |  |  |  |  |  |  |  |  |  |  |  |  | 1 | 0 |
| 21 | *Bx* | *CxTx* |  |  |  |  |  |  |  |  |  |  |  |  |  |  |  |  | 0 | 2 |
| 22 | *Bx* | *C4Tx* |  |  |  |  |  |  |  |  |  |  |  |  |  |  |  |  | 1 | 0 |
| 23 | *Bx* | *CxTx* |  |  |  |  |  |  |  |  |  |  |  |  |  |  |  |  | 1 | 2 |
| 25 | *Bx* | *C4Tx* |  |  |  |  |  |  |  |  |  |  |  |  |  |  |  |  | 1 | 0 |
| 27 | *Bx* | *CxTx* |  |  |  |  |  |  |  |  |  |  |  |  |  |  |  |  | 0 | 3 |
| 28 | *Bx* | *CxT4* |  |  |  |  |  |  |  |  |  |  |  |  |  |  |  |  | 2 | 1 |
| 31 | *Bx* | *CxTx* |  |  |  |  |  |  |  |  |  |  |  |  |  |  |  |  | 0 | 2 |
| 33 | *Bx* | *CxTx* |  |  |  |  |  |  |  |  |  |  |  |  |  |  |  |  | 2 | 1 |
| 35 | *Bx* | *CxTx* |  |  |  |  |  |  |  |  |  |  |  |  |  |  |  |  | 0 | 1 |
| 37 | *Bx* | *CxTx* |  |  |  |  |  |  |  |  |  |  |  |  |  |  |  |  | 0 | 1 |
| 38 | *Bx* | *CxTx* |  |  |  |  |  |  |  |  |  |  |  |  |  |  |  |  | 0 | 1 |
| 44 | *Bx* | *CxTx* |  |  |  |  |  |  |  |  |  |  |  |  |  |  |  |  | 2 | 1 |
| 50 | *Bx* | *CxTx* |  |  |  |  |  |  |  |  |  |  |  |  |  |  |  |  | 0 | 2 |
| 57 | *Bx* | *CxTx* |  |  |  |  |  |  |  |  |  |  |  |  |  |  |  |  | 2 | 0 |
| 62 | *Bx* | *CxTx* |  |  |  |  |  |  |  |  |  |  |  |  |  |  |  |  | 2 | 0 |
| 63 | *Bx* | *CxTx* |  |  |  |  |  |  |  |  |  |  |  |  |  |  |  |  | 0 | 1 |
| 64 | *Bx* | *CxTx* |  |  |  |  |  |  |  |  |  |  |  |  |  |  |  |  | 2 | 0 |
| 68 | *Bx* | *CxT4* |  |  |  |  |  |  |  |  |  |  |  |  |  |  |  |  | 1 | 1 |
| 69 | *Bx* | *CxT4* |  |  |  |  |  |  |  |  |  |  |  |  |  |  |  |  | 1 | 1 |
| 70 | *Bx* | *C4T4* |  |  |  |  |  |  |  |  |  |  |  |  |  |  |  |  | 1 | 1 |
| 71 | *Bx* | *C4Tx* |  |  |  |  |  |  |  |  |  |  |  |  |  |  |  |  | 4 | 10 |
| 73 | *Bx* | *C4T4* |  |  |  |  |  |  |  |  |  |  |  |  |  |  |  |  | 3 | 4 |
| 78 | *Bx* | *CxT4* |  |  |  |  |  |  |  |  |  |  |  |  |  |  |  |  | 1 | 0 |
| 81 | *Bx* | *C4T4* |  |  |  |  |  |  |  |  |  |  |  |  |  |  |  |  | 1 | 0 |
| 86 | *Bx* | *CxT4* |  |  |  |  |  |  |  |  |  |  |  |  |  |  |  |  | 0 | 2 |
| 87 | *Bx* | *C4T4* |  |  |  |  |  |  |  |  |  |  |  |  |  |  |  |  | 1 | 1 |
| 90 | *Bx* | *C4Tx* |  |  |  |  |  |  |  |  |  |  |  |  |  |  |  |  | 5 | 2 |
| 91 | *Bx* | *C4Tx* |  |  |  |  |  |  |  |  |  |  |  |  |  |  |  |  | 0 | 1 |
| 92 | *Bx* | *CxTx* |  |  |  |  |  |  |  |  |  |  |  |  |  |  |  |  | 0 | 1 |
| 94 | *Bx* | *C4Tx* |  |  |  |  |  |  |  |  |  |  |  |  |  |  |  |  | 0 | 1 |
| 104 | *Bx* | *CxTx* |  |  |  |  |  |  |  |  |  |  |  |  |  |  |  |  | 3 | 0 |
| 112 | *Bx* | *C4Tx* |  |  |  |  |  |  |  |  |  |  |  |  |  |  |  |  | 1 | 0 |
| 113 | *Bx* | *C4Tx* |  |  |  |  |  |  |  |  |  |  |  |  |  |  |  |  | 0 | 2 |
| 117 | *Bx* | *CxTx* |  |  |  |  |  |  |  |  |  |  |  |  |  |  |  |  | 1 | 0 |
| 118 | *Bx* | *CxT4* |  |  |  |  |  |  |  |  |  |  |  |  |  |  |  |  | 1 | 1 |
| 150 | *Bx* | *CxT4* |  |  |  |  |  |  |  |  |  |  |  |  |  |  |  |  | 3 | 0 |
| 159 | *Bx* | *C4Tx* |  |  |  |  |  |  |  |  |  |  |  |  |  |  |  |  | 1 | 1 |
| 163 | *Bx* | *CxTx* |  |  |  |  |  |  |  |  |  |  |  |  |  |  |  |  | 0 | 1 |
| 171 | *Bx* | *CxTx* |  |  |  |  |  |  |  |  |  |  |  |  |  |  |  |  | 0 | 1 |
| 178 | *Bx* | *CxTx* |  |  |  |  |  |  |  |  |  |  |  |  |  |  |  |  | 1 | 0 |
| 184 | *Bx* | *C4Tx* |  |  |  |  |  |  |  |  |  |  |  |  |  |  |  |  | 1 | 0 |
| 187 | *Bx* | *CxTx* |  |  |  |  |  |  |  |  |  |  |  |  |  |  |  |  | 1 | 0 |
| 191 | *Bx* | *CxTx* |  |  |  |  |  |  |  |  |  |  |  |  |  |  |  |  | 1 | 1 |
| 192 | *Bx* | *CxTx* |  |  |  |  |  |  |  |  |  |  |  |  |  |  |  |  | 1 | 0 |
| 202 | *Bx* | *CxTx* |  |  |  |  |  |  |  |  |  |  |  |  |  |  |  |  | 1 | 1 |
| 205 | *Bx* | *CxTx* |  |  |  |  |  |  |  |  |  |  |  |  |  |  |  |  | 0 | 1 |
| 233 | *Bx* | *CxTx* |  |  |  |  |  |  |  |  |  |  |  |  |  |  |  |  | 2 | 1 |
| 260 | *Bx* | *CxTx* |  |  |  |  |  |  |  |  |  |  |  |  |  |  |  |  | 0 | 1 |
| 269 | *Bx* | *CxTx* |  |  |  |  |  |  |  |  |  |  |  |  |  |  |  |  | 0 | 1 |
| 275 | *Bx* | *CxTx* |  |  |  |  |  |  |  |  |  |  |  |  |  |  |  |  | 0 | 1 |
| 277 | *Bx* | *CxTx* |  |  |  |  |  |  |  |  |  |  |  |  |  |  |  |  | 0 | 1 |
| 331 | *Bx* | *CxTx* |  |  |  |  |  |  |  |  |  |  |  |  |  |  |  |  | 1 | 0 |
| 336 | *Bx* | *CxTx* |  |  |  |  |  |  |  |  |  |  |  |  |  |  |  |  | 2 | 0 |
| 337 | *Bx* | *CxTx* |  |  |  |  |  |  |  |  |  |  |  |  |  |  |  |  | 0 | 1 |
| 339 | *Bx* | *CxTx* |  |  |  |  |  |  |  |  |  |  |  |  |  |  |  |  | 0 | 1 |
| 367 | *Bx* | *C4Tx* |  |  |  |  |  |  |  |  |  |  |  |  |  |  |  |  | 1 | 0 |
| 370 | *Bx* | *CxTx* |  |  |  |  |  |  |  |  |  |  |  |  |  |  |  |  | 1 | 0 |
| 381 | *Bx* | *CxTx* |  |  |  |  |  |  |  |  |  |  |  |  |  |  |  |  | 0 | 1 |
| 382 | *Bx* | *C4Tx* |  |  |  |  |  |  |  |  |  |  |  |  |  |  |  |  | 0 | 2 |
| 384 | *Bx* | *CxTx* |  |  |  |  |  |  |  |  |  |  |  |  |  |  |  |  | 2 | 0 |
| 393 | *Bx* | *CxTx* |  |  |  |  |  |  |  |  |  |  |  |  |  |  |  |  | 0 | 1 |
| 397 | *Bx* | *CxTx* |  |  |  |  |  |  |  |  |  |  |  |  |  |  |  |  | 1 | 0 |
| 401 | *Bx* | *C4Tx* |  |  |  |  |  |  |  |  |  |  |  |  |  |  |  |  | 0 | 1 |
| 404 | *Bx* | *C4Tx* |  |  |  |  |  |  |  |  |  |  |  |  |  |  |  |  | 1 | 0 |
| 416 | *Bx* | *C4Tx* |  |  |  |  |  |  |  |  |  |  |  |  |  |  |  |  | 1 | 0 |
| 463 | *Bx* | *CxTx* |  |  |  |  |  |  |  |  |  |  |  |  |  |  |  |  | 1 | 0 |
| 466 | *Bx* | *CxTx* |  |  |  |  |  |  |  |  |  |  |  |  |  |  |  |  | 0 | 1 |
| 522 | *Bx* | *C4Tx* |  |  |  |  |  |  |  |  |  |  |  |  |  |  |  |  | 0 | 1 |
| 554 | *Bx* | *CxTx* |  |  |  |  |  |  |  |  |  |  |  |  |  |  |  |  | 0 | 1 |
| 557 | *Bx* | *CxTx* |  |  |  |  |  |  |  |  |  |  |  |  |  |  |  |  | 0 | 1 |
| 566 | *Bx* | *CxTx* |  |  |  |  |  |  |  |  |  |  |  |  |  |  |  |  | 0 | 1 |
| 567 | *Bx* | *CxTx* |  |  |  |  |  |  |  |  |  |  |  |  |  |  |  |  | 0 | 1 |
| 585 | *Bx* | *CxTx* |  |  |  |  |  |  |  |  |  |  |  |  |  |  |  |  | 0 | 1 |
| 600 | *Bx* | *CxTx* |  |  |  |  |  |  |  |  |  |  |  |  |  |  |  |  | 0 | 1 |
| 602 | *Bx* | *CxTx* |  |  |  |  |  |  |  |  |  |  |  |  |  |  |  |  | 0 | 1 |
| 691 | *Bx* | *CxTx* |  |  |  |  |  |  |  |  |  |  |  |  |  |  |  |  | 0 | 1 |
| *Total* | | | | | | | | | | | | | | | | | | | 181 | 181 |

**Supplementary Table S2:** KIR profile in patients with AML and healthy controls. Gray and white cells indicate the presence and absence of related genes, respectively.
